# Acute dyskinesia, myoclonus, and akathisa in an adolescent male abusing quetiapine via nasal insufflation: a case study

**DOI:** 10.1186/1471-2431-13-187

**Published:** 2013-11-16

**Authors:** Mathew George, Maya Haasz, Alvaro Coronado, Steven Salhanick, Lindsey Korbel, Joseph P Kitzmiller

**Affiliations:** 1 Department of Pediatrics, St. Barnabas Hospital, 4432 3rd avenue, Bronx, NY 10457, USA; 2 Department of Emergency Medicine, Beth Israel Deaconess medical center, 330 Brookline Avenue, Boston, MA 02215, USA; 3 College of Medicine, Ohio State University, 333 West 10th Avenue, Columbus, OH 43210, USA

**Keywords:** Tardive dyskinesia, Medication abuse, Quetiapine insufflation

## Abstract

**Background:**

Although the benefits of antipsychotic pharmacotherapy can be pronounced, many patients develop unwanted adverse effects including a variety of movement disorders. Compared with the traditional antipsychotics, the atypical antipsychotics have a decreased risk for associated movement disorders. Drug-induced movement disorders can occur, however, and the risk of adverse events can increase significantly when medications are abused.

**Case presentation:**

We describe the case of a 13-year-old male who presented to an emergency department with acute movement disorders after nasal insufflation of crushed quetiapine. The patient was admitted and successfully treated for neuroleptic toxicity with intravenous antihistamine pharmacotherapy. His primary care provider and psychiatrist were notified of the abuse, quetiapine was discontinued, and the patient was discharged and referred to a drug and alcohol awareness and abuse program.

**Conclusions:**

The abuse of quetiapine has unfortunately become more common. This unique case report of acute movement disorders following nasal insufflation of quetiapine highlights the need for heightened vigilance when prescribing quetiapine and for increased awareness and education regarding medication-abuse.

## Background

Quetiapine is an atypical antipsychotic, and its indications include psychosis, mood disorders, and bipolar disorder. It is intended for oral administration with total daily doses up to 800 mg in adults. Quetiapine has good efficacy, but also has some potential for abuse (see Table 1). Routes of abuse include insufflation and intravenous entries.

**Table 1:** Case report of quetiapine abuse

**First author**	**Year**	**Patient demographics**	**Description of quetiapine abuse**	**Other relevant details**
Fischer [1]	2010	53-year-old male	Unknown amount, orally	Alcohol abuse
Paparrigopoulos [2]	2008	48-year-old male	1000 mg/day orally	Alcohol/benzodiazepine dependence
Murphy [3]	2008	29-year-old male	Unknown amount, orally	Feigned psychotic symptoms
Reeves [4]	2007	49-year-old male	800 mg/day orally	Alcohol/benzodiazepine abuse
		23-year-old male	2400 mg/day, orally	Benzodiazepine dependence
		39-year-old male	800 mg/day, orally	Exaggerated bipolar symptoms
Pinta [5]	2007	39-year-old male	600 mg/day, orally	Opiate abuse; demanded treatment with quetiapine
Morin [6]	2007	28-year-old female	Unknown amount, insufflation	Polysubstance abuse
Waters [7]	2007	33-year-old male	400-800 mg, intravenously	Polysubstance dependence including benzodiazepines
Hussain [8]	2005	34-year-old female	600 mg, intravenously	Polysubstance abuse, borderline personality disorder

Quetiapine has been associated infrequently with tardive dyskinesia [9-11] and with acute movements disorders including myoclonus [12-14], dystonia [15], parkinsonism [16] and akathisia [17]. Drug-induced movement disorders have also been associated with cases of abuse [18]; however, most subjects were either psychotic inpatients or incarcerated individuals. This case is unique in that it involves an adolescent abusing quetiapine, via nasal insufflation, in an out-patient setting.

## Case presentation

A 13-year old male presented to an ED with complaints of “frequent eye blinking” and reoccurring episodes of “stiffening and abnormal movements of the hands and neck” and “flickering of the upper lips” that began 24 hours prior to his arrival. About one week prior to presenting at the ED, the patient had been discharged from a psychiatric hospital, and his discharge medications for his mood disorder (Mood Disorder Not Otherwise Specified) included quetiapine 500 milligrams (mg) by mouth (PO) daily (qd) and valproic acid (VPA) 500 mg PO at bedtime (qhs). His dosing regimen of quetiapine for the three months before that hospitalization had been 100 mg qd, and he had not previously been prescribed VPA.

The patient reported having insufflated two crushed tablets of quetaipine 500 mg on four separate occasions in the previous forty-eight hours. His desire to experience euphoria motivated him to abuse his prescription quetiapine. He reported having not taken his prescribed VPA in three days and also reported that he had not recently used any other medications, supplements, or illicit drugs. His symptoms began two hours after the last insufflation of quetiapine, the episodes of excessive eye-blinking and lip-flickering were intermittent, and the twitching of his eyelids was continuous.

At the time of presentation, the patient was fully alert and had a Glascow Coma Scale of 15. He was afebrile, tachycardic (115 beats/minute), tachypnic (18 respirations/minute), and had normal oxygen saturation without supplemental oxygen. Physical exam abnormalities included only active twitching of both upper eyelids and bilateral dilated pupils (4/5). During observation in the emergency department, the patient had two myoclonic episodes of the extremities and intense flickering of the eyelids that the patient reported were associated with him turning his head to the right. The episodes lasted about two minutes, and the patient was alert and oriented during the episodes. The patient reported feeling restless and had a constant desire to walk. Results from a 10-panel urine toxicology screen performed at admission were negative for common drugs of abuse, and lorazepam 1.5 mg intravenous (IV) was given to relax the patient. His restlessness worsened, however, and he was admitted for observation and treatment of neuroleptic toxicity. Diphenhydramine 50 mg IV was administered, and he was in stable condition within 24 hours. Quetiapine was discontinued, and the patient was discharged and referred for substance abuse evaluation and treatment. His primary care provider and psychiatrist were notified regarding the medication abuse.

## Conclusions

We report a case of acute movement disorders in an adolescent that likely resulted from quetiapine abuse (nasal insufflation of crushed tablets). Many patients embellish or malinger to obtain quetiapine, and its abuse is not uncommon [8,19]. Quetiapine tablets can be crushed into powder, and many abusers will either insufflate the powder or will solubilize the powder and inject it intravenously. Abusers choose these routes of administration in order to experience more rapid onset of quetiapine’s anxiolytic effects. The toxicokinetics of intranasal insufflation of quetiapine have not been fully characterized, but this route of administration undoubtedly leads to significant acute levels of quetiapine in the central nervous system. This sudden substantial exposure is likely responsible for the acute episodes of dyskinesia, myoclonus and akathisa described in this case. The movement disorders described in this case were not likely related to use of VPA because their associations with VPA are extremely rare [20] and the patient had not been exposed to VPA (half-life 9–16 hours) in several days.

Exposure to other drugs of abuse (e.g., cocaine, phencyclidine) can cause acute movement disorders; however, the urine toxicology results and the patient report do not suggest that other drugs of abuse were involved in this case report.

Per Hill’s Criteria of Causation [21], one can reasonably conclude that this patient’s dyskinesia was related to quetiapine exposure. According to Narnajo’s Adverse Drug Reaction (ADR) scale [22] for this case, the score was 6, indicating probable cause (> 9 = definite ADR, 5–8 = probable ADR, 1–4 = possible ADR 0 = doubtful ADR). The quick response to anticholinergic pharmacotherapy is also suggestive of acute neuroleptic-associated dyskinesia. Limitations of our report include a lack of serum quetiapine measurements. This report, however, highlights the need for increased prescribing vigilance and abuse-potential awareness.

## Consent

Written informed consent was obtained from the patient’s legal guardian and from the patient for publication of this case report. A copy of the written consent is available for review by the Editor of this journal.

## Abbreviations

GABA: Gamma-aminobutyric acid; Mg: Milligrams; PO: By mouth; qd: Daily; qhs: At bedtime; IV: Intravenous; VPA: Valproic acid; ADR: Adverse drug reaction.

## Competing interests

The authors declare that they do not have any financial or any non-financial (political, personal, religious, ideological, academic, intellectual, commercial or any other) competing interests regarding this manuscript.

## Authors’ contributions

MG, MH, AC, and SS were responsible for the medical care of the patient and assisted in the literature review and drafting of the Case Report. LK and JPK were responsible for the literature review and drafting of the Case Report. All authors contributed to the Conclusions, and all authors approve the final manuscript.

## Pre-publication history

The pre-publication history for this paper can be accessed here:

http://www.biomedcentral.com/1471-2431/13/187/prepub
